# Tracking Candidemia Trends and Antifungal Resistance Patterns across Europe: An In-Depth Analysis of Surveillance Systems and Surveillance Studies

**DOI:** 10.3390/jof10100685

**Published:** 2024-09-29

**Authors:** Karin Odoj, Jacopo Garlasco, Maria Diletta Pezzani, Cristina Magnabosco, Diego Ortiz, Federica Manco, Liliana Galia, Sarah K. Foster, Fabiana Arieti, Evelina Tacconelli

**Affiliations:** 1 Division of Infectious Diseases, Department of Internal Medicine I, University Hospital Tübingen, 72076 Tübingen, Germany; karin.odoj@med.uni-tuebingen.de (K.O.); diego.ortiz@med.uni-tuebingen.de (D.O.); sarahkat519@gmail.com (S.K.F.); 2 Infectious Disease Unit, Department of Diagnostic and Public Health, University of Verona, 37129 Verona, Italy; jacopo.garlasco@univr.it (J.G.); cristina.magnabosco@univr.it (C.M.); federica.manco@univr.it (F.M.); liliana83galia@gmail.com (L.G.); fabiana.arieti@univr.it (F.A.); evelina.tacconelli@univr.it (E.T.)

**Keywords:** Candidemia, antifungal drug resistance, surveillance, epidemiology, European data, public health reporting

## Abstract

Background: The WHO fungal priority list classifies *Candida* species as critical and high-priority pathogens, and the WHO GLASS fungi initiative seeks to establish a standardised global framework for antifungal resistance monitoring. We aimed to review resistance rates and antifungal resistance patterns across European surveillance systems and studies in response to these recent calls for action. Methods: A systematic review of national and international surveillance systems and peer-reviewed surveillance studies available up to June 2024 was conducted. Descriptive and trend analyses were performed on surveillance data reporting resistance to different antifungals in *Candida* spp. Results: In total, 6 national surveillance systems and 28 studies from 13 countries provided candidemia resistance data, mostly about the *C. albicans*, *C. glabrata* and *C. parapsilosis* complex. Azole resistance was most frequently reported (6/6 surveillance systems and 27/28 studies) with the highest resistance rate, especially for *C. glabrata*, in Croatia (100%, 28/28 isolates) and Slovenia (85.7%, 82/96) and *C. parapsilosis* in Croatia (80.6%, 54/67) and Italy (72.6%, 106/146). Echinocandin and polyene resistance rates were nearly zero. The number of isolates included in the surveillance systems increased over the years, particularly for *C. albicans* (+40–60 isolates/year), *C. glabrata*, and *C. parapsilosis* (+15–30 isolates/year). No surveillance system or study reported resistance data for *C. auris*. Pooled data from national surveillance revealed a decreasing trend in azole resistance in *C. albicans* and *C. glabrata*. The increasing azole-resistance trend in *C. parapsilosis* disappeared after adjusting for between-country heterogeneity. Overall, echinocandin and polyene resistance trends appeared relatively stable. Conclusions: Awareness of antifungal resistance is growing, but further actions are needed to strengthen surveillance capacity and knowledge-sharing networks across Europe.

## 1. Introduction

Candidemia, a bloodstream infection caused by *Candida* species, represents a significant healthcare challenge globally, with Europe bearing a substantial burden of this infection [1,2].

In Europe, the distribution and resistance rates of *Candida* species in candidemia are highly heterogeneous. Compared to studies from the early 2000s, the latest European Confederation of Medical Mycology (ECMM) *Candida* study revealed a decrease in the proportion of *C. albicans* (56.4%) and an increase in *C. glabrata* (13.6%). However, at the country level, the highest proportion of *C. albicans* was observed in Austria (77%), *C. parapsilosis* in Italy and Turkey (24–26%), and *C. glabrata* in the Czech Republic, France, and the UK (25–33%). One of the most pressing issues in the management of candidemia is the emergence of antifungal resistance, driven by the inappropriate use of antifungals, widespread prophylactic use in high-risk populations, and environmental factors within healthcare facilities [3,4]. Currently, approved antifungals against candidiasis belong to three main antimicrobial classes that was used to treat invasive fungal infections: azoles (e.g., fluconazole, voriconazole, itraconazole, posaconazole), echinocandins (e.g., anidulafungin, caspofungin, micafungin), and polyenes (e.g., amphotericin B). The ECMM study confirmed low resistance rates for echinocandins but highlighted a high rate of fluconazole resistance (12%) in *C. glabrata*. Particularly concerning was the overall fluconazole resistance rate of 24% across Greece, Italy, and Turkey [5]. In addition, the emergence of *C. auris* in Europe has led to a series of concerning outbreaks, reflecting its rapid spread and multidrug-resistant nature. The United Kingdom has documented over 200 cases since 2015, while Spain reported 591 cases between 2020 and 2021. Italy experienced inter-facility spreads with 277 cases in 2022. Further sporadic outbreaks have been reported across Europe, highlighting the widespread and persistent nature of this pathogen. [6,7].

The surveillance of candidemia is crucial for understanding its epidemiology, trends, and the emergence of resistant strains. In 2019, the World Health Organization (WHO) introduced a protocol to include *Candida* spp. in its Global Antimicrobial Resistance and Use Surveillance System (GLASS). Additionally, it developed a surveillance module (GLASS-FUNGI) to monitor antimicrobial resistance in invasive fungal infections, although data from this initiative are not yet publicly available [8]. At the European level, collaborative efforts such as the European Antimicrobial Resistance Surveillance Network (EARS-Net) and the ECMM support the harmonisation of surveillance methodologies and the exchange of data and best practices among member states [9,10]. These systems often rely on national or regional networks of hospitals, laboratories, and public health agencies to collect standardised data on isolates, patient demographics, clinical outcomes, and antifungal susceptibility profiles [11]. In October 2022, the WHO released its first fungal priority pathogens list (WHO FPPL), designating four *Candida* species as critical (*C. auris* and *C. albicans*) and high priority (*C. tropicalis* and *C. parapsilosis*). This document aims to prompt public health authorities to take action to enhance diagnostics, monitor antifungal resistance, and advance research efforts together with policy interventions [12].

In 2022, we published an initial evaluation of the status of resistance surveillance in *Candida* spp. blood isolates using publicly accessible surveillance systems and epidemiological studies across Europe. Our findings indicated that resistance surveillance in candidemia remains underemphasised, with only five countries reporting resistance among *Candida* blood isolates and 13 studies assessing resistance. Additionally, the variability in reporting, data collection and definitions hindered the possibility of an accurate burden assessment [13].

Building on our previous work, this article aims to provide an updated overview and synthesis of resistance data in candidemia and related antifungal resistance patterns from European surveillance systems and published surveillance studies considering the initiatives mentioned above.

## 2. Materials and Methods

### 2.1. Data and Search Strategy

All data used in this study were collected as part of the EPI-Net project activities [14]. Established in 2015, EPI-Net provides a platform to facilitate the use of epidemiological data and optimise the surveillance of antimicrobial resistance (AMR) and healthcare-associated infections (HAIs): further details are available at the EPI-Net website [15]. A review of publicly available national and international surveillance systems was conducted to access and analyse epidemiological data on resistant *Candida* species from blood cultures in Europe, as previously described [13]. The review protocol is available on the EPI-Net website. In brief, data from national surveillance systems reporting resistance patterns against azoles (fluconazole, voriconazole, itraconazole), echinocandins (anidulafungin, caspofungin, micafungin), and polyenes (amphotericin B) among six *Candida* species (*C. albicans*, *C. glabrata*, *C. parapsilosis*, *C. tropicalis*, *C. krusei*, and *C. auris*) were analysed. The availability of surveillance data have been regularly mapped, and data have been collected according to the accessibility of the specific reports provided by each country; therefore, the data may be based on non-random or non-systematic sampling methods. Moreover, epidemiological surveillance studies on resistant *Candida* strains were identified in the PubMed database (Tables S1 and S3).

### 2.2. Inclusion Criteria for National Surveillance Systems and Multicentre Surveillance Studies

Only surveillance systems providing or intending to provide data on an annual basis were included. Surveillance systems without publicly available information or with information older than 2015 only were excluded, as well as single-centre data or reports with data retrieved from the same national surveillance systems already included. Surveillance studies were included if published between 1 January 2005 and 30 June 2024, conducted in at least two centres and for at least 12 consecutive months. Studies including centres in two or more countries were included if they reported resistance data disaggregated by country. Data were independently collected by two reviewers, and disagreements were resolved by discussion or by requesting an evaluation from a third reviewer.

### 2.3. Data Analysis and Statistics

Descriptive statistics were provided for each drug–pathogen combination in terms of resistance rates. Resistance rates were defined as the percentage of *Candida* species that were resistant to the defined antifungal agent among the tested isolates (e.g., percentage of *C. albicans* isolates resistant to azoles among all *C. albicans* isolates from blood samples). The median resistance rate was calculated based on the individual resistance rates for each *Candida* species provided by either all national surveillance systems or all surveillance studies in the respective country. The EUCAST and CLSI standards were primarily used as reference guidelines (see Supplementary Table S3 for detailed information). Trend analyses were performed on the number of isolates and on resistance rates for the data obtained from the national surveillance systems. Trends on the number of isolates were evaluated through linear regression based on the ordinary least squares method. For resistance rates, national trends were built using a generalised linear model of the binomial family, allowing for weighting by a number of isolates. The overall trends were evaluated first using the same method (by pooling data from all countries in each year) and, secondly, when possible, using a partially Bayesian multilevel model based on generalised linear mixed-effect regressions of the binomial family. This allowed for the presence of a fixed effect from time (i.e., the trend) and random effects from countries (in consideration of the expectably high heterogeneity); *p*-values < 0.05 were considered statistically significant. The figures were plotted, and statistical analyses were conducted using R software (version 4.3.1); in particular, the “blme” package was used to build the trend models [16,17].

### 2.4. Ethics

As our research did not involve human or animal subjects, ethical approval was not required. This study was based exclusively on aggregate data retrieved from published surveillance reports and epidemiological studies, which ensured full conformity of the study with the European Regulations in terms of Data Protection and Privacy (GDPR 2016/679).

## 3. Results

### 3.1. Candidemia Surveillance in Europe

**Figure 1:**
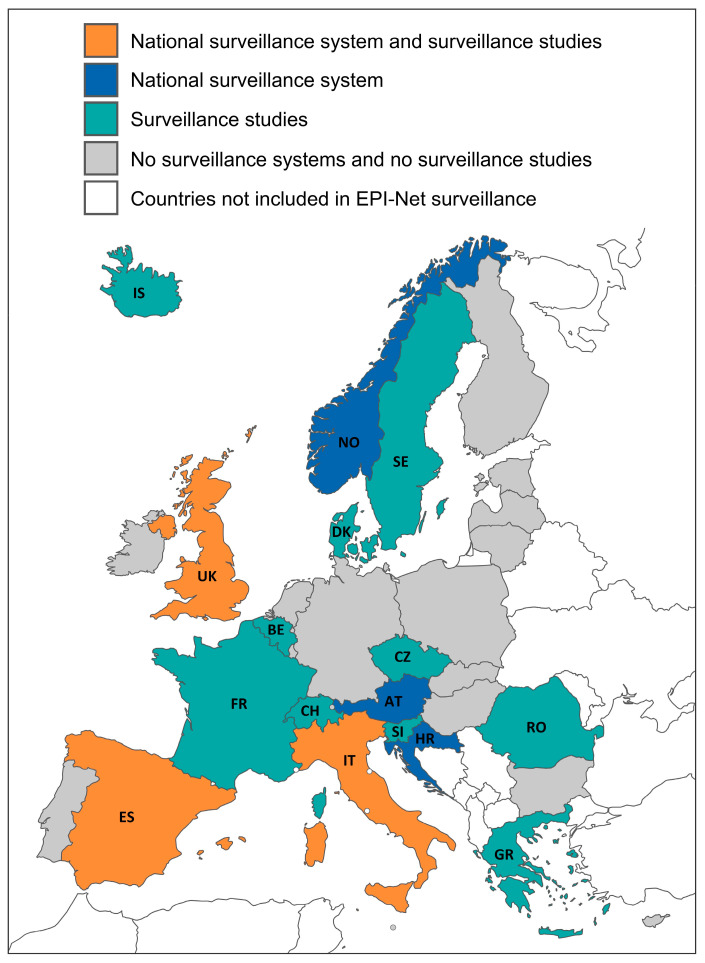
Countries with available surveillance data of resistance in candidemia. Blue: countries providing data from national surveillance systems; green: countries providing data from surveillance studies; orange: countries providing data from national surveillance systems and surveillance studies; grey: countries without available resistance data in *Candida* spp. or data that do not meet our inclusion criteria; white: countries not included in EPI-Net surveillance scope; and grey circles: European microstates (Liechtenstein, Malta, Monaco, San Marino, and Vatican City).

Of the 32 European countries reviewed, 6 countries (Austria, Croatia, Italy, Norway, Spain, United Kingdom) provided data from national surveillance systems on antifungal resistance in *Candida* blood cultures on a regular basis (Figure 1, Table S2). Compared to our previous assessment of candidemia surveillance in Europe, we found one additional country (i.e., Croatia) with newly available, up-to-date surveillance data on *Candida* resistance. In the remaining 26 countries, we could not find resistance data for the individual *Candida* species. Resistance data on candidemia from the international surveillance systems (EARS-Net, FWD-Net, HAI-Net, GLASS) are still unavailable [13].

In addition to the data from national surveillance systems, we identified a total of 28 surveillance studies (out of 1361 screened studies) from 13 countries (Belgium, Czech Republic, Denmark, France, Greece, Iceland, Italy, Romania, Slovenia, Spain, Sweden, Switzerland, and the United Kingdom) that met our inclusion criteria and reported *Candida* spp. resistance data. The evaluation periods for the included studies spanned from the years 2000 to 2022 (Table S3).

#### 3.1.1. Representation of *Candida* Species and Resistance Patterns in Surveillance Studies

The number of countries and surveillance studies reporting resistance to different antifungals (azoles, echinocandins, polyenes) varied between *Candida* species. All countries with *Candida* surveillance studies reported azole resistance in *C. albicans*, *C. glabrata*, and *C. parapsilosis*. Within the included studies, resistant *C. albicans* was the most reported species, with the highest number of tested isolates considering all antifungal classes (23,115) included in these studies, followed by *C. glabrata* and *C. parapsilosis* (Table 1). No surveillance study on *C. auris* resistance met our inclusion criteria.

A comparative analysis from the most recently evaluated isolates in surveillance studies revealed a large variation in *Candida* species distribution and resistance rates across the countries (Table 2) [5,18,19,20,21,22,23,24,25,26,27,28,29,30,31,32,33,34,35,36,37,38,39,40,41,42,43,44].

Azole resistance rates were the highest in *C. glabrata*, with a median resistance rate of 11.0%, followed by *C. tropicalis* and *C. parapsilosis* (with median resistance rates 6% and 4%, respectively). *C. albicans* and *C. krusei* showed resistance rates close to or equal to zero in most cases (Table 2a). The highest azole resistance rates in surveillance studies including 20 or more isolates were reported for *C. krusei* in Denmark (88.5%, 46/52 isolates), for *C. glabrata* in Slovenia (85.7%, 82/96 isolates), and for *C. parapsilosis* in Italy (72.6%, 106/146 isolates).

Resistance rates for echinocandins were mainly present in *C. glabrata*, with a median resistance rate of 0.5%. Six countries reported resistance to echinocandins in *C. glabrata* (Belgium, Denmark, Greece, Slovenia, Sweden, Switzerland), although with low resistance rates, ranging from 1.0% (Belgium, 1/97 isolates) to 3.7% (Sweden, 3/83 isolates, Table 2b). Median echinocandin resistance rates for *C. albicans*, *C. parapsilosis*, *C. tropicalis*, and *C. krusei* were all equal to 0%.

Polyene resistance rates were very low (Table 2c). The highest values in surveillance studies, including 20 or more isolates, were reported for *C. glabrata* in Slovenia (11.2%, 11/96 isolates) and France (9.2%, 6/65 isolates).

#### 3.1.2. Representation of *Candida* Species and Resistance Patterns in National Surveillance Systems

Similar to the surveillance studies, data for the different classes of antifungal agents and *Candida* strains showed considerable heterogeneity across countries. All six national surveillance systems reported data on azole-resistant *C. albicans* (Table 3). Five national surveillance systems provided resistance data for all three major antifungal classes in *C. albicans* and *C. parapsilosis.* Four surveillance systems supplied data on *C. glabrata* resistance, whereas three systems reported on resistance in *C. tropicalis* for all three antifungal classes. Only one surveillance system (Croatia) provided data on *C. krusei*’s resistance against echinocandins and polyenes, while no data were available from any national surveillance system for resistant *C. auris* strains. The detailed list of countries with surveillance systems monitoring resistance for respective antifungal—*Candida* species combinations is reported in Table 3.

A quantitative analysis of the most recent year of available surveillance data from national surveillance systems revealed a considerable variation in the number of tested isolates between countries. Overall, *C. albicans* had the highest number of tested isolates across all surveillances (1227 for azoles, 925 for echinocandins and 1109 for polyenes), with the United Kingdom contributing the most to these numbers. *C. glabrata* was the second most common species reported (528 for azoles, 481 for echinocandins and 608 for polyenes), followed by *C. parapsilosis* (353 for azoles, 280 for echinocandins and 339 for polyenes). Tested isolates of *C. tropicalis* (39 for all antifungals) and *C. krusei* (6 for echinocandins and polyenes) were very low (Table 4).

Resistance rates from the last year of available national candidemia surveillance data showed substantial heterogeneity between different countries and antifungal classes (Table 5). Azole resistance rates in *Candida* species ranged from 0% in *C. albicans* (Austria, Norway, Spain), *C. parapsilosis* (Austria and Norway), and *C. tropicalis* (Norway) to 100% in *C. glabrata* (Croatia, 28/28 isolates). Notably, *C. glabrata* and *C. tropicalis* showed the highest median resistance rates for azoles (15.8% and 5.9%, respectively), and *C. albicans* showed the lowest ones (0.7%). For *C. parapsilosis*, the highest azole resistance rates were found in Croatia (80.6%, 54/67 tested isolates) and Spain (43.5%, 10/23 isolates).

In contrast, resistance rates to echinocandins showed a narrower range, from 0% found in several different *Candida* species (in Austria, Croatia, Norway, Spain, and United Kingdom) to 15.4% in *C. parapsilosis* (in Spain, 2/13 isolates). The highest rates for *C. tropicalis* and *C. albicans* were observed in Austria at 5.9% (1/17 isolates) and 5.8% (11/191 isolates), respectively.

Polyene resistance rates were almost zero for *C. albicans*, *C. glabrata*, *C. parapsilosis*, and *C. tropicalis* in most surveillance systems (Austria, Croatia, Norway, Spain). High resistance rates to polyenes were reported by only one surveillance system (Croatia) with a low number of isolates for *C. krusei* (50%, 3/6 tested isolates) and *C. tropicalis* (20%, 2/10 isolates). No other countries reported polyene resistance in *C. krusei.* And no countries reported any resistance data for *C. auris.* Only one national surveillance system from the United Kingdom reported the incidence of candidemia, with an incidence of 4.0 per 100,000 inhabitants in 2022.

### 3.2. Trends in Isolates Included in Surveillances and Antifungal Resistance Rates across Europe

**Figure 2:**
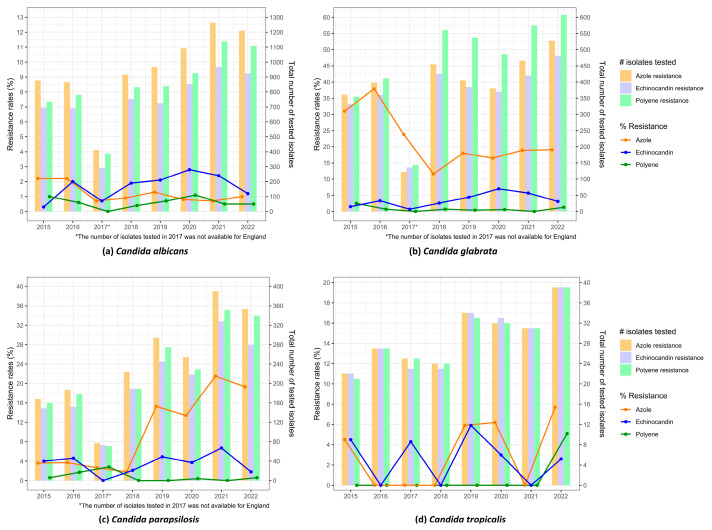
Pooled antifungal resistance rates from all national surveillance systems in (**a**) *C. albicans*; (**b**) *C. glabrata*; (**c**) *C. parapsilosis*; and (**d**) *C. tropicalis.* Resistance rates are shown as lines corresponding to the scale on the left *y*-axis, and the total number of tested isolates are shown as bars corresponding to the *y*-axis on the right.

The rates of antifungal resistance and the numbers of tested isolates over the years from all six countries with national surveillance systems for the different antifungal classes (azoles, echinocandins and polyenes) in *C. albicans*, *C. glabrata*, *C. parapsilosis* and *C. tropicalis* are illustrated in Figure 2.

The resistance rates to echinocandins and polyenes remained relatively stable across all *Candida* species (Figure 2a–d). In contrast, regarding azole resistance rates, the visual inspection of the plots suggested the presence of a downward trend over the years for *C. glabrata* (Figure 2b) and an increase for *C. parapsilosis* from 2019 onwards (Figure 2c). However, while the crude model with pooled isolates confirmed this result (β = −0.128 and β = 0.329 for *C. glabrata* and *C. parapsilosis*, respectively, both *p* < 0.001), the model accounting for country-related heterogeneity failed to find any significant trends for these pathogens (*p* = 0.765 and *p* = 0.812 respectively, Table 6). In *C. tropicalis*, there was a degree of variability in resistance levels over time, even due to the very low number of isolates included in the surveillance systems (Figure 2d), although no significant trends were observed (Table 5).

Considering the number of tested isolates, a general trend towards stability or a slight increase was observed over the years. These increases were more relevant for species with a higher number of isolates (*C. albicans*, *C. glabrata* and *C. parapsilosis*) and were often statistically significant (details in Table 6 and Figure 2a–d).

An analysis of the trends in resistance rates over the years within each single country showed an increasing trend for echinocandin-resistant *C. albicans* in Austria (Supplementary Table S4) and a significantly decreasing trend for azole-resistant *C. albicans* and *C. glabrata* in the United Kingdom. In the other countries, no significant changes in resistance rates were observed over the years. Trends in resistance rates of *Candida* isolates from surveillance systems are displayed in Table 7.

## 4. Discussion

Candidemia represents a significant healthcare challenge worldwide, with an incidence rate of 3–5 per 100,000 persons in the general population, a global annual incidence of around 750,000 cases/year and a mortality rate between 40% and 60%, depending on factors such as the patient’s condition, the Candida species involved, or the promptness of the initiation of antifungal therapy [45].

The analysis of data from national surveillance systems showed increasing trends in the number of tested isolates for all pathogens. This might be a consequence of the higher incidence of candidemia or the increased awareness of antifungal resistance in *Candida* species [1,46,47]. Considering antifungal resistance rates, generally decreasing trends in azole resistance were detected for *C. albicans* and *C. glabrata*, and conversely, an increase in azole resistance was found in the last few years for *C. parapsilosis*. However, it is important to carefully consider the significant variation in data availability across different surveillance systems. For instance, including countries with very high resistance rates in recent years (e.g., Croatia for azole-resistant *C. parapsilosis*, Table S4) or the presence of a significant trend in countries with larger number of isolates (e.g., the United Kingdom) may affect the overall analysis of resistance rates.

Our findings, from both national surveillance systems and surveillance studies, showed that the most reported species remained *C. albicans*, *C. glabrata*, and *C. parapsilosis* and resistance to azoles was most frequently reported. Azole resistance exhibited the highest resistance rates: both national surveillance systems and studies confirmed high azole resistance in *C. glabrata* (e.g., 100% in Croatia, 85.7% in Slovenia) and *C. parapsilosis* (e.g., 80.6% in Croatia and 72.6% in Italy). Except for some cases with very low numbers of isolates, echinocandin and polyene resistance rates were lower, with values generally ranging between 0% and 5.9% in national surveillance systems and between 0% and 4.2% in studies (with some exceptions for polyene-resistant *C. glabrata*, e.g., France 9.2% and Slovenia 11.2%, and echinocandin-resistant *C. parapsilosis*, e.g., Sweden 19.4%). Pooled data from national surveillance systems pointed to a decreasing trend in azole resistance for *C. albicans* and *C. glabrata* (*p* ≤ 0.001) and an increase in azole-resistant *C. parapsilosis* (*p* < 0.001). Data from Austria showed an increasing trend in echinocandin-resistant *C. albicans.* However, trends in antifungal resistance for echinocandins and polyenes seemed relatively stable across most *Candida* species. For three countries, Italy, Spain and United Kingdom, data were available from both surveillance systems and studies. In these cases, we found discrepancies between the two sources, as studies usually reported lower or null resistance rates. For instance, surveillance systems in the United Kingdom reported resistance rates of 14.5% (54/372 isolates) for *C. glabrata* to azoles in 2022, while a study collecting data from 2018 to 2022 reported no resistant isolates. This stresses the importance of state-coordinated surveillance systems as a more complete source of data. Nevertheless, on account of the lack of surveillance systems or publicly available data, studies still represent a valuable tool to monitor resistance. For instance, studies have reported high resistance rates to azoles for *C. krusei* in Denmark, for *C. parapsilosis* in Greece and Italy, and for *C. glabrata* in Slovenia [31,32,38,41].

Our analyses corroborate the reports of increasing fluconazole resistance in *C. parapsilosis* globally in recent years [48]. In Europe, Arendrup et al. identified *C. albicans*, *C. glabrata*, and *C. parapsilosis* as the most common species associated with candidemia, highlighting concerning fluconazole resistance rates in *C. glabrata* and *C. parapsilosis* [5]. Increased azole resistance in *C. parapsilosis* has been documented in Spain, with resistant genotypes circulating in the community, regardless of azole exposure history [49,50]. Similarly, high azole resistance in non-albicans species has been reported in a three-year observational study in Croatia [51]. France also reported fluconazole-resistant *C. parapsilosis* outbreaks in 2021 and 2023 [52,53]. Although echinocandin resistance remains relatively uncommon compared to azoles, there is increased reporting of resistance in *C. parapsilosis* and *C. glabrata* [54,55,56,57]. Such reports warrant attention as acquired resistance for both species is driven by selective pressure due to the increased use of these antifungal classes [57,58,59,60].

Among the limitations of this study, some are linked to access to data on invasive Candida infections: the EPI-Net platform attempts to include data from all national surveillance systems publicly available but fails to include single-centre data or networks that monitor candidemia with an internal protocol without providing periodical reports on public platforms. For example, France and Germany have well-established surveillance systems (i.e., the RESSIF Network [61] and the AReST Project [62], respectively), yet the absence of periodic, publicly available reports limits the acquisition of updated figures from two of the most populous countries in continental Europe [63]. Secondly, a limitation may arise from the limited number of isolates, which, for instance, may affect the computation of trend models: not coincidentally, most trends resulting from the simple linear model were not confirmed when accounting for heterogeneity. Indeed, the very low numbers reported by some surveillance systems also cast doubts on the representativeness of the respective samples for whole countries: while some settings (e.g., Italy, Scotland) are particularly lacking in recent data from 2019-2020 onwards, maybe even as an aftermath of the surveillance disruption induced by COVID-19, for some other countries (e.g., Spain, Croatia) the numbers are constantly low throughout the years (Table S4). Moreover, except for ESPAUR in the United Kingdom, surveillance systems related to Candida fail to report antifungal consumption data, thus preventing exploration of any correlation between inappropriate antifungal usage and resistance rates. Eventually, among the surveillance systems, only a minority (Austria and partially England) consider some demographic factors such as age and gender.

Our work highlights the still limited surveillance coverage for candidemia across Europe, with publicly available data coming from only six national surveillance systems out of 32 countries. Additionally, the lack of standardisation in methods for the identification of *Candida* species and resistance patterns among these surveillance systems hinders the comparison of reported data. One of the frequent weaknesses of surveillance systems is the missing information on patient characteristics, the spatial or regional origins of the cases, and antifungal prescription practices [64]. Therefore, more granular data might be useful to target possible actions in lowering the incidence of candidemia. Finally, significant knowledge gaps exist in countries lacking such surveillance systems. For example, studies indicate some of the highest resistance rates to azoles (*C. glabrata*) and polyenes (*C. glabrata* and *C. krusei*). However, the most recent data available are from 2012, leaving current resistance rates unknown [32]. Notably, no current surveillance system is reporting on *C. auris*. In the case of studies, we found that although many publications provided information on *Candida* resistance, most of them did not provide complete data, hindering the use of these works for further analysis.

Besides GLASS-FUNGI, other valuable efforts to collect and standardise international antifungal resistance data include the SENTRY Antimicrobial Surveillance Program (39 countries, 1997–ongoing) [65,66]. To our knowledge, there are currently no other international surveillance systems with publicly available data on antifungal resistance in Europe. The FPPL and GLASS-FUNGI initiatives are expected to motivate national authorities to establish antifungal resistance surveillance systems. However, it is crucial for these authorities to ensure the necessary conditions for the timely development and implementation of these systems.

## 5. Conclusions

Our findings align with current knowledge as *C. albicans* resistance rates are commonly reported by established surveillance systems. However, the surveillance of *C. glabrata* and *C. parapsilosis* is intensifying globally, reflecting the rising incidence of candidemia caused by non-albicans species. Azole resistance rates were particularly high in *C. glabrata*, *C. tropicalis*, and *C. parapsilosis*, likely indicating the selection of resistant strains and the spread of resistance genes in *C. parapsilosis* and *C. tropicalis* [59,60]. Despite *C. auris* being listed as a critical priority pathogen in the WHO FPPL, with its resistance being increasingly reported in the literature, it has not yet been reported in any national surveillance system.

There is a growing recognition of the importance of integrating candidemia surveillance into broader antimicrobial resistance surveillance frameworks and public health agendas. International initiatives and multidisciplinary collaboration are essential to address major challenges such as variations in surveillance practices and data completeness across different countries, the underreporting of cases, limited availability of molecular typing techniques for strain characterisation, and to ensure the comparability of data between regions and over time.

In conclusion, while significant strides have been made in establishing candidemia surveillance infrastructure across Europe, ongoing investments, innovation, and collaboration are needed to address existing gaps and adapt to evolving epidemiological trends and antimicrobial resistance threats. By strengthening surveillance capacity and knowledge-sharing networks, Europe can better respond to the complex and dynamic nature of candidemia and mitigate its impact on public health.

## Figures and Tables

**Table 1:** Countries with surveillance studies reporting data on antifungal resistance in different *Candida* species. No data were available for *C. auris*.

Pathogen	Antifungal	N° of Countries Reporting Data	N° of Surveillance Studies	N° of Isolates
*C. albicans*	Azole	13 (BE; CH; CZ; DK; ES; FR; GR; IS; IT; RO; SI; SE; UK)	25	8595
	Echinocandin	12 (BE; CH; CZ; DK; ES; FR; GR; IS; IT; SI; SE; UK)	24	6066
	Polyene	12 (BE; CH; CZ; DK; ES; FR; GR; IS; IT; SI; SE; UK)	23	8454
*C. glabrata*	Azole	13 (BE; CH; CZ; DK; ES; FR; GR; IS; IT; RO; SI; SE; UK)	25	3572
	Echinocandin	12 (BE; CH; CZ; DK; ES; FR; GR; IS; IT; SI; SE; UK)	23	3001
	Polyene	11 (BE; CH; CZ; DK; ES; FR; IS; IT; SI; SE; UK)	21	2986
*C. parapsilosis*	Azole	13 (BE; CH; CZ; DK; ES; FR; GR; IS; IT; RO; SI; SE; UK)	27	3593
	Echinocandin	12 (BE; CH; CZ; DK; ES; FR; GR; IS; IT; SI; SE; UK)	21	1381
	Polyene	11 (BE; CH; CZ; DK; ES; FR; IS; IT; SI; SE; UK)	20	1113
*C. tropicalis*	Azole	11 (BE; CH; CZ; DK; ES; GR; IS; IT; SI; SE; UK)	22	1143
	Echinocandin	11 (BE; CH; CZ; DK; ES; GR; IS; IT; SI; SE; UK)	23	670
	Polyene	10 (BE; CH; CZ; DK; ES; IS; IT; SI; SE; UK)	20	679
*C. krusei*	Azole	6 (CH; DK; ES; IT; SI; SE)	12	383
	Echinocandin	7 (CH; DK; ES; GR; IT; SI; SE)	14	342
	Polyene	6 (CH; DK; ES; IT; SI; SE)	12	391

BE: Belgium; CZ: Czech Republic; DK: Denmark; FR: France; GR: Greece; IS: Iceland; IT: Italy; RO: Romania; SI: Slovenia; ES: Spain; SE: Sweden; CH: Switzerland; and UK: United Kingdom.

**Table 2:** Resistance rates for different *Candida* species from the most recent data collected in surveillance studies in each country ^§^. Asterisks (* and **) indicate resistance rates corresponding to specific years.

Country	Surveillance Period	*C. albicans*	*C. glabrata*	*C. parapsilosis*	*C. tropicalis*	*C. krusei*
**(a) Azole Resistance** N° resistant isolates/N° total isolates (% resistance)						
Belgium	2013–2014; * 2018–2022	**7/179 (3.9)**	**11/97 (11.3)**	3/5 (60) *	**4/20 (20.0)**	-
Czech Republic	2012–2015; * 2018–2022	0/28 (0.0)	0/0 (0.0) *	1/6 (16.7) *	**1/8 (12.5)**	-
Denmark	2016–2018	**3/602 (0.5)**	**49/460 (10.7)**	**1/60 (1.6)**	**3/70 (4.0)**	**46/52 (88.5)**
France	2014–2018	0/135 (0.0)	**8/66 (12.1)**	0/41 (0.0)	-	-
Greece	2009–2018; * 2018–2022	**49/1883 (3.0)**	1/2 (50%) *	**441/2216 (20.0)**	**23/373 (6.0)**	-
Iceland	2000–2011	0/124 (0.0)	1/36 (2.8)	0/11 (0.0)	**1/28 (3.6)**	-
Italy	2020–2021; * 2018–2022; ** 2016–2017	**4/104 (3.8)**	1/8 (12.5) *	**106/146 (72.6)**	**1/9 (11.1)**	0/10 (0.0) **
Romania	2010–2011	**1/57 (1.8)**	**4/25 (16.0)**	0/59 (0.0)	-	-
Slovenia	2001–2012	**1/272 (0.4)**	**82/96 (85.7)**	**1/35 (2.7)**	0/19 (0.0)	0/11 (0.0)
Spain	2005–2006	0/97 (0.0)	**1/27 (3.7)**	0/34 (0.0)	0/30 (0.0)	0/7 (0.0)
Sweden	2015–2016; * 2018–2022	**5/200 (2.5)**	0/3 (0.0) *	1/14 (7.1) *	0/15 (0.0)	**5/7 (71.4)**
Switzerland	2014–2018	0/330 (0.0)	**16/176 (9.0)**	**2/50 (4.0)**	**5/45 (11.0)**	0/11 (0.0)
United Kingdom	2006–2017; * 2018–2022	**1/46 (2.2)**	0/11 (0.0) *	2/6 (33.3) *	0/4 (0.0)	-
**Median resistance rate (%)**		**0.45**	**11.00**	**4.00**	**6.00**	0.00
**(b) Echinocandin Resistance** N° resistant isolates/N° total isolates (% resistance)						
Belgium	2013–2014	0/179 (0.0)	**1/97 (1.0)**	0/35(0.0)	0/20 (0.0)	-
Czech Republic	2012–2015	**1/28 (3.6)**	0/10 (0.0)	0/5 (0.0)	**1/8 (12.5)**	-
Denmark	2016–2018	0/608 (0.0)	**6/454 (1.3)**	0/61 (0.0)	0/75 (0.0)	**3/69 (4.2)**
France	2014–2018	0/135 (0.0) ^‡^	0/67 (0.0) ^‡^	0/41 (0.0) ^‡^	-	-
Greece	2009–2018	0/724 (0.0)	**7/203 (3.0)**	0/396 (0.0)	0/75 (0.0)	0/33 (0.0)
Iceland	2000–2011	0/41 (0.0) ^‡^	0/14 (0.0) ^‡^	0/5 (0.0) ^‡^	0/7 (0.0)*^‡^*	-
Italy	2020–2021; ** 2016–2017	**3/86 (3.5)**	0/28 (0.0)	0/127 (0.0)	0/8 (0.0)	0/10 (0.0) **
Slovenia	2001–2012	**1/177 (0.6) ^‡^**	**2/65 (2.9) ^‡^**	0/20 (0.0) ^‡^	0/14 (0.0) ^‡^	0/7 (0.0) ^‡^
Spain	2005–2006	0/97 (0.0) ^‡^	0/27 (0.0) ^‡^	0/34 (0.0) ^‡^	0/30 (0.0) ^‡^	0/7 (0.0) ^‡^
Sweden	2015–2016	**1/199 (0.5)**	**3/81 (3.7)**	**7/36 (19.4)**	**1/12 (8.3)**	**3/9 (33.3)**
Switzerland	2014–2018	**2/212 (1.0)**	**1/124 (1.0)**	0/39 (0.0)	0/32 (0.0)	0/15 (0.0)
United Kingdom	2006–2017; * 2012–2013	0/46 (0.0) ^†^	0/17 (0.0) ^†^	0/29 (0.0) *	0/4 (0.0) ^‡^	-
**Median resistance rate (%)**		0.00	**0.50**	0.00	0.00	0.00
**(c) Polyene Resistance** N° resistant isolates/N° total isolates (% resistance)						
Belgium	2013–2014	0/179 (0.0)	0/97 (0.0)	0/35 (0.0)	0/20 (0.0)	-
Czech Republic	2012–2015	0/28 (0.0)	0/10 (0.0)	0/5 (0.0)	0/8 (0.0)	-
Denmark	2016–2018	0/608 (0.0)	0/460 (0.0)	0/61 (0.0)	0/75 (0.0)	0/72 (0.0)
France	2014–2018	0/135 (0.0)	**6/65 (9.2)**	0/41 (0.0)	-	-
Greece	2009–2018	**49/1883 (3.0)**	-	-	-	-
Iceland	2000–2011	0/124 (0.0)	0/36 (0.0)	0/11 (0.0)	0/28 (0.0)	-
Italy	2020–2021; * 2011–2015	0/44 (0.0) *	0/16 (0.0) *	1/35 (2.9) *	0/13 (0.0) *	0/14 (0.0) *
Slovenia	2001–2012	0/272 (0.0)	**11/96 (11.2)**	**1/35 (2.7)**	0/19 (0.0)	**6/11 (54.5)**
Spain	2005–2006	0/97 (0.0)	0/27 (0.0)	0/34 (0.0)	0/30 (0.0)	0/7 (0.0)
Sweden	2015–2016	0/235 (0.0)	0/81 (0.0)	0/37 (0.0)	0/13 (0.0)	0/13 (0.0)
Switzerland	2014–2018	0/214 (0.0)	0/147 (0.0)	**1/40 (2.5)**	0/35 (0.0)	0/16 (0.0)
United Kingdom	2006–2017	0/46 (0.0)	0/17 (0.0)	0/23 (0.0)	0/4 (0.0)	-
**Median resistance rate (%)**		0.0	0.0	0.0	0.0	0.0

^§^ Azole resistance refers to fluconazole except for *C. krusei*, where resistance refers to voriconazole due to its intrinsic resistance to fluconazole; echinocandin resistance refers to anidulafungin where not otherwise indicated (**^‡^** caspofungin, **^†^** micafungin); and polyene resistance refers to amphotericin B. Resistance rates > 0 are marked in bold.

**Table 3:** Countries with surveillance systems reporting data on antifungal resistance in different *Candida* species. No data were available for *C. auris*.

Candida Species	Antifungal	N° of Countries with Surveillance Systems
*C. albicans*	Azole	6 (AT; HR; IT; NO; ES; UK)
	Echinocandin	5 (AT; HR; NO; ES; UK)
	Polyene	5 (AT; HR; NO; ES; UK)
*C. glabrata*	Azole	4 (AT; HR; NO; UK)
	Echinocandin	4 (AT; HR; NO; UK)
	Polyene	4 (AT; HR; NO; UK)
*C. parapsilosis*	Azole	5 (AT; HR; NO; ES; UK)
	Echinocandin	5 (AT; HR; NO; ES; UK)
	Polyene	5 (AT; UK; NO; ES; HR)
*C. tropicalis*	Azole	3 (AT; NO; HR)
	Echinocandin	3 (AT; NO; HR)
	Polyene	3 (AT; NO; HR)
*C. krusei*	Azole	-
	Echinocandin	1 (HR)
	Polyene	1 (HR)

AT: Austria; HR: Croatia; IT: Italy; NO: Norway; ES: Spain; and UK: United Kingdom.

**Table 4:** The number of isolates tested for each *Candida* species from the most recent year of available surveillance data from national surveillance systems in respective countries. * No data available for *C. krusei*.

Antifungal	*Candida* Species	Austria 2022	Croatia 2022	Italy 2019	Norway 2022	Spain 2022	United Kingdom 2022	N° Total Isolates
Azole	*C. albicans*	191	67	17	139	17	796	1227
	*C. glabrata*	87	28	-	41	-	372	528
	*C. parapsilosis*	28	67	-	17	23	218	353
	*C. tropicalis*	17	10	-	12	-	-	39
	*C. krusei*	-	- *	-	-	-	-	-
Echinocandin	*C. albicans*	191	67	-	139	10	518	925
	*C. glabrata*	87	28	-	41	-	325	481
	*C. parapsilosis*	28	67	-	17	13	155	280
	*C. tropicalis*	17	10	-	12	-	-	39
	*C. krusei*	-	6	-	-	-	-	6
Polyene	*C. albicans*	191	67	-	15	139	697	1109
	*C. glabrata*	87	28	-	41	-	452	608
	*C. parapsilosis*	28	67	-	17	22	205	339
	*C. tropicalis*	17	10	-	12	-	-	39
	*C. krusei*	-	6	-	-	-	-	6

**Table 5:** Resistance rates for each *Candida* species from the most recent year of available surveillance data in respective countries ^§^.

Antifungal	*Candida* Species	Austria 2022	Croatia 2022	Italy 2019 ^‡^	Norway 2022	Spain 2022 ^‡^	United Kingdom 2022 ^†^	Median Resistance Rate (%)
Azole	*C. albicans*	0/191 (0.0)	2/67 (3.0)	2/17 (11.8)	0/139 (0.0)	0/17 (0.0)	10/796 (1.3)	0.7
	*C. glabrata*	12/87 (13.8)	28/28 (100.0)	-	7/41 (17.1)	-	54/372 (14.5)	15.8
	*C. parapsilosis*	0/28 (0.0)	54/67 (80.6)	-	0/17 (0.0)	10/23 (43.5)	4/218 (1.8)	1.8
	*C. tropicalis*	1/17 (5.9)	2/10 (20.0)	-	0/12 (0.0)	-	-	5.9
Echinocandin	*C. albicans*	11/191 (5.8)	0/67 (0.0)	-	0/139 (0.0)	0/10 (0.0)	0/518 (0.0)	0.0
	*C. glabrata*	0/87 (0.0)	0/28 (0.0)	-	2/41 (4.9)	-	13/325 (4.0)	2.0
	*C. parapsilosis*	1/28 (3.7)	1/67 (1.6)	-	0/17 (0.0)	2/13 (15.4)	1/155 (0.6)	1.6
	*C. tropicalis*	1/17 (5.9)	0/10 (0.0)	-	0/12 (0.0)	-	-	0.0
	*C. krusei*	-	0/6 (0.0)	-	-	-	-	0.0
Polyene	*C. albicans*	0/191 (0.0)	0/67 (0.0)	-	0/15 (0.0)	0/139 (0.0)	5/697 (0.7)	0.0
	*C. glabrata*	0/87 (0.0)	0/28 (0.0)	-	0/41 (0.0)	-	8/452 (1.8)	0.0
	*C. parapsilosis*	0/28 (0.0)	1/67 (1.5)	-	0/17 (0.0)	0/22 (0.0)	1/205 (0.5)	0.0
	*C. tropicalis*	0/17 (0.0)	2/10 (20.0)	-	0/12 (0.0)	-	-	0.0
	*C. krusei*	-	3/6 (50.0)	-	-	-	-	50.0 *

^§^ Data are shown in the form of N° resistant isolates/N° total isolates (% resistance). ^‡^ Data from intensive care unit isolates; ^†^ data from England only; and * single value from one surveillance system.

**Table 6:** Trends in the total number of *Candida* isolates monitored by national surveillance systems ^§^.

Antifungal	*Candida* Species	Isolate Number Growth per Year (β)	*p*-Value
Azole	*C. albicans*	**58.0**	**0.004**
	*C. glabrata*	16.7	0.059
	*C. parapsilosis*	**29.7**	**0.004**
	*C. tropicalis*	**2.0**	**0.015**
Echinocandin	*C. albicans*	**39.6**	**0.007**
	*C. glabrata*	**15.5**	**0.030**
	*C. parapsilosis*	**23.3**	**0.006**
	*C. tropicalis*	**2.0**	**0.018**
	*C. krusei*	1.1	0.062
Polyene	*C. albicans*	**56.8**	**0.004**
	*C. glabrata*	**31.8**	**0.008**
	*C. parapsilosis*	**27.3**	**0.005**
	*C. tropicalis*	**2.1**	**0.011**
	*C. krusei*	1.1	0.062

^§^ Results from the years 2015 to 2022 are shown; *p*-values < 0.05 were considered statistically significant and are marked in bold. The number of isolates for 2017 was not reported in England; hence, this year was not considered for the computation of trends in isolates’ number.

**Table 7:** Trends in resistance rates of *Candida* isolates monitored by national surveillance systems ^§^.

		(Generalised Linear Model (Not Accounting for Heterogeneity)			Bayesian Multilevel Mixed-Effects Model (Accounting for Heterogeneity)		
		(Without Accounting for Heterogeneity)			(Accounting for Heterogeneity)		
Antifungal	*Candida* Species	Slope (Trend)	*p*-Value	Trend	Slope (Trend)	*p*-Value	Trend
Azole	*C. albicans*	**−0.151**	**0.001**	**↘**	−0.046	0.735	↔
	*C. glabrata*	**−0.128**	**<0.001**	**↘**	−0.122	0.765	↔
	*C. parapsilosis*	**0.329**	**<0.001**	**↗**	0.035	0.812	↔
	*C. tropicalis*	0.219	0.213	↔	0.227	0.530	↔
Echinocandin	*C. albicans*	0.085	0.053	↔	−0.050	0.708	↔
	*C. glabrata*	0.121	0.006	↔	0.003	0.982	↔
	*C. parapsilosis*	0.017	0.768	↔	−0.477	0.134	↔
	*C. tropicalis*	−0.043	0.811	↔	−0.525	0.554	↔
Polyene	*C. albicans*	−0.030	0.652	↔	**−0.041**	**<0.001**	**↘**
	*C. glabrata*	−0.143	0.077	↔	0.022	0.982	↔
	*C. parapsilosis*	−0.248	0.082	↔	-	-	-

^§^ Trends include all subsets of isolates for which the resistance rate was known in the years between 2015 and 2022. For each antifungal drug–pathogen *Candida* species combination, the three columns on the left represent the trend evaluated by the generalised linear model after crude pooling of the isolates from all countries, while the three columns on the right show the trends computed by a multilevel model with a random-effect part considering the heterogeneity between countries. *p*-values < 0.05 were considered statistically significant and are marked in bold. Polyene resistance in *C. tropicalis* was constantly equal to zero throughout the years; thus, no trend could be computed with any model.

## Data Availability

Data are available on the EPI-Net website: https://epi-net.eu/.

## Supplementary Materials

The following supporting information can be downloaded at https://www.mdpi.com/article/10.3390/jof10100685/s1. Table S1: Search terms used in to identify national surveillance systems and epidemiological surveillance studies; Table S2: National surveillance systems in Europe, providing antifungal resistance data for *Candida* spp.; Table S3: Microbiological testing and reference guidelines adopted in the studies to test Candida species; Table S4: Number of isolates and resistance rates from different *Candida* species from all years of available national surveillance data in the respective countries for azole resistances, echinocandin resistances and polyene resistances.
