# Supplementary material for: Tracking Candidemia Trends and Antifungal Resistance Patterns across Europe: An In-Depth Analysis of Surveillance Systems and Surveillance Studies
Source: J Fungi (Basel). 2024 Sep 29;10(10):685. doi: 10.3390/jof10100685 (PMC11514733; doi:10.3390/jof10100685)
Supplement: Supplementary file 1 [file jof-10-00685-s001.zip › Supplementary Table S1.pdf]

Supplementary Table S1. Search terms used in to identify national surveillance systems and epidemiological surveillance studies

	National surveillance systems	Surveillance studies
**Source**	Google	PubMed
**Search terms***	'Antimicrobial resistance'<br>'Antibiotic resistance'<br>'Multidrug resistance'<br>'Antifungal resistance'<br><br>'Candidemia',<br>Mycosis',<br>'Fungi',<br><br>'Surveillance'<br>'Monitoring'<br>'Infection'<br><br>'Nosocomial'<br>'Healthcare'<br>'Hospital'<br>'Healthcare-associated infection'<br>'Hospital-associated infection'	'Antifungal resistance '<br>'Multidrug resistance '<br><br>AND<br><br>'Surveillance'<br>'Epidemiology'<br>'Monitoring'<br>'Incidence'<br>'Prevalence'<br><br>AND<br><br>'Epidemiology', 'Epidemiology'<br>'Fungemia', 'Fungaemia'<br>'Candidemia', 'Candidaemia', 'Candida'<br>'Bacteraemia', 'Bacteremia', 'Bloodstream infection'

*Search terms for national surveillance systems were used in the local languages of the European countries
