# Supplementary material for: Tracking Candidemia Trends and Antifungal Resistance Patterns across Europe: An In-Depth Analysis of Surveillance Systems and Surveillance Studies
Source: J Fungi (Basel). 2024 Sep 29;10(10):685. doi: 10.3390/jof10100685 (PMC11514733; doi:10.3390/jof10100685)
Supplement: Supplementary file 1 [file jof-10-00685-s001.zip › Supplementary Table S2.pdf]

Supplementary Table S2. National surveillance systems in Europe, providing antifungal resistance data for *Candida* spp.

National Surveillance Systems		
**Country**	**Surveillance System**	**Link**
**Austria**	AURES	https://www.sozialministerium.at/Themen/Gesundheit/Antimikrobielle-Resistenzen/Ma%C3%9Fnahmen-im-Bereich-antimikrobieller-Resistenzen-(AMR)-/Aktionspl%C3%A4ne-und-Bericht-.html#aures-der-oesterreichische-antibiotikaresistenz-bericht-01-1
**Croatia**	ISKRA	https://iskra.bfm.hr/otpornost-bakterija-na-antibiotike-u-hrvatskoj/
**Italy**	GiViTi	https://giviti.marionegri.it/report_infezioni/
**Norway**	NORM	https://www.fhi.no/hn/helseregistre-og-registre/norm/om-norm/
**Spain**	ENVIN	http://hws.vhebron.net/envin-helics/
**United Kingdom (Scotland)**	PHS	https://publichealthscotland.scot/our-areas-of-work/health-protection/
**United Kingdom (England)**	ESPAUR	https://www.gov.uk/government/publications/english-surveillance-programme-antimicrobial-utilisation-and-resistance-espaur-report
