# Supplementary material for: Tracking Candidemia Trends and Antifungal Resistance Patterns across Europe: An In-Depth Analysis of Surveillance Systems and Surveillance Studies
Source: J Fungi (Basel). 2024 Sep 29;10(10):685. doi: 10.3390/jof10100685 (PMC11514733; doi:10.3390/jof10100685)
Supplement: Supplementary file 1 [file jof-10-00685-s001.zip › Supplementary Table S3.pdf]

Supplementary Table S3. Microbiological testing and reference guidelines adopted in the studies to test *Candida* species. AMB: Amphtericin; AND: anidulafungin; CAS: caspofungin; FLU: fluconazole; ITR: itraconazole; MIC: micafungin; POS: posaconazole; VOR: voriconazole;

Author	Country	Setting/Year	Microbiological Testing	Reference Guidelines	*Species*	Antifungal tested	DOI
**Adam K.M., et al., (2021)**	Switzerland	Hospital 2004-2008	Sensititre YeastOne/Etest/ MALDI-TOF/Vitek	CLSI M60-Ed2	*C. albicans, C. glabrata, C. parapsilosis, C. krusei, C. tropicalis*	AND, FLU, VOR, AMB	10.1093/ofid/ofab471
**Arendrup M.C., et al. (2023)**	Sweden, UK, Belgium, Czech Republic, Italy, Greece	Hospital 2018-2022	broth microdiluition EUCAST Edef 7.3	EUCAST	*C. parapsilosis, C. glabrata*	FLU	10.1016/j.jinf.2023.08.001
**Arendrup M.C., et al. (2013)**	Denmark	Hospital 2010-2011	broth microdiluition EUCAST Edef 7.2/ E-test	EUCAST technical note 2011/ CLSI M27-A3 (2008)	*C. albicans, C. dubliniensis, C. glabrata C. krusei, C. parapsilosis, C. tropicalis*	AMB, AND, CAS, FLU, VOR, POS, ITR	10.1111/1469-0691.12212
**Astvad K.M.T., et al. (2018)**	Denmark	Hospital 2012 -2015	broth microdiluition EUCAST Edef 7.3/ E-test	EUCAST	*C. albicans, C. dubliniensis, C. glabrata C. krusei, C. parapsilosis, C. tropicalis*	AND, FLU, VOR, AMB, MIC	10.1128/JCM.01564-17
**Arendrup M.C., et al. (2011)**	Denmark	Hospital 2004-2009	broth microdiluition EUCAST Edef 7.3/ E-test	EUCAST/ CLSI M27-A3 (2008)	*C. albicans, C. dubliniensis, C. glabrata C. krusei, C. parapsilosis, C. tropicalis*	AMB, AND, CAS, FLU, VOR, POS, ITR	10.1128/JCM.01811-10
**Asmundsdottir L.R., et al. (2013)**	Iceland	Hospital 2000-2011	broth microdilution E-test method (AB bioMérieux, Solna, Sweden)/ RPMI 1640 agar medium with 2% glucose (Sigma-Aldrich, St. Louis, MO)	CLSI/ CLSI M27-S3	*C. albicans, C. glabrata, C. tropicalis, C. dubliniensis, C. parapsilosis*	AMB, FLU, ITR, VOR, CAS	10.1128/JCM.02566-12
**Bassetti M., et al. (2007)**	Italy	Hospital 2004-2005	broth microdiluition RPMI 1640	CLSI M27-A2	*C. albicans, Candida spp.*	FLU, ITR, VOR, AMB, CAS	10.1016/j.diagmicrobio.2007.01.005
**Bassetti M., et al, (2013)**	Italy, Spain	Hospital 2008-2010	broth microdilution SensititreYeastOne	CLSI M27-S4 (2012)	*C. albicans, C. glabrata, C. parapsilosis complex, C. tropicalis*	FLU, CAS	10.1128/JCM.01998-13
**Desnos-Ollivier M., et al (2018)**	France	Hospital 2003-2013	broth microdilution EUCAST Edef 7.2	EUCAST EDEF 7.2	*C. parapsilosis*	POS, FLU, VOR	10.1007/s11046-017-0224-7

**Ericsson J., et al. (2012)**	Sweden	Laboratory 2005-2006	Etest (ABbioMerieux, Solna, Sweden)	EUCAST vers. 1.0 (2007)/ CLSI	*C. albicans, C. glabrata, C. parapsilosis, C. dubliniensis, C. tropicalis, C. lusitaniae, C. krusei*	AMB, FLU, VOR, CAS	10.1111/1469-0691.12111
**Florez C., et al. (2009)**	Spain	Hospital, Laboratory 2005-2006	SensititreYeastOne/ Etest	CLSI M44-S1/CLSI M27-A3	*C. albicans, C. parapsilosis, C. tropicalis, C. glabrata, C. krusei, C. famata, C. guilliermondii*	AMB, FLU, ITR, VOR, CAS	10.1016/j.eimc.2008.09.013
**Klingspor L., et al. (2018)**	Sweden	Laboratory 2015-2016	SensititreYeastOne/ E-test (bioMérieux) /Vitek2/	EUCAST vers. 8.0 (2015)	*C. albicans, C. glabrata, C. parapsilosis complex, C. tropicalis, C. krusei, C. dubliniensis, C. lusitaniae, C. kefyr, C. lipolytica, C. magnoliae, C. pelliculosa, C. sake, C. orthopsilosis*	AND, FLU, AMB, VOR	10.1111/myc.12816
**Kocmanová I., et al. (2018)**	Czech Republic	Laboratory 2012-2015	Broth microdilution EUCAST Edef 7.3	EUCAST	*C. albicans, C. glabrata, C. parapsilosis, C. tropicalis*	FLU, AND, AMB	PMID: 30157661
**Luzzati R., et al. (2016)**	Italy	Hospital 2011-2013	SensititreYeastOne	EUCAST vers. 6.1 (2013)	*C. albicans, C. glabrata, C. parapsilosis, C. tropicalis*	FLU, CAS, AMB	10.1007/s15010-016-0924-9
**Mamali V., et al.(2022)**	Greece	Hospital 2009-2018	SensititreYeastOne/ E-test (bioMérieux) /Vitek2/MTS	CLSI M60/epidemiological cut-off values (ECVs)	*C. albicans, C. glabrata, C. parapsilosis* complex, *C. tropicalis, C. krusei*	FLU, AND, AMB	10.3390/jof8020116
**Matos T., et al. (2021)**	Slovenia	Hospital 2001-2012	E-test (bioMérieux) /Vitek2	CLSI M27-A3(for amphotericin B if MIC was equal or below 1 mg/L as suggested by Pfaller et al.)	*C. albicans, C. glabrata, C. parapsilosis, C. krusei, C. tropicalis*	FLU, VOR, CAS, AMB	10.1111/myc.13278
**Minea B., et al. (2015)**	Romania	Hospital 2010-2011	Broth microdilution EUCAST vers. 6.1	EDef 7.1/ EUCAST EDef 7.2	*C. albicans, C. glabrata, C. parapsilosis*	FLU	10.1007/s10096-014-2240-6
**Posteraro B., et al. (2020)**	Italy	Hospital 2011-2015	Sensititre YeastOne	CLSI M27-A3 (then replaced by the CLSI document M60)	*C. albicans, C. glabrata, C. parapsilosis, C. krusei, C. tropicalis*	FLU, VOR, AND, AMB	10.1111/myc.13130

**Prigitano A., et al. (2016)**	Italy	Hospital, Laboratory 2014-2015	Sensititre YeastOne colorimetric plates (Trek Diagnostic Systems, Cleveland, OH, USA)/E-test	CLSI/epidemiological cut-off values (modal MIC + one doubling dilution)	*C. albicans, C. glabrata, C. parapsilosis, C. tropicalis, C. krusei*	AND, CAS, MIC, FLU, VOR, ITR, POS, AMB	10.1007/s15010-016-0951-6
**Prigitano A., et al. (2020)**	Italy	Hospital 2016-2017	Sensititre YeastOne (SYO, Thermo Scientific Trek Diagnostic Systems, East Grinstead, UK) / Vitek 2	CLSI M27-A3	*C. albicans, C. glabrata, C. parapsilosis, C. tropicalis, C. krusei*	AND, FLU, ITR, AMB	10.1016/j.mycmed.2019.100906
**Prigitano A., et al.(2023)**	Italy	Hospital 2020-2021	Sensititre Yeast One/Vitek 2	CLSI/EUCAST E.Def 7.3, E.Def 9.4 and E.Def 11.0	*C. albicans, C. glabrata, C. parapsilosis, C. tropicalis*	FLU, ITR, POS, VOR, AND, CAS, AMB	10.3390/jof9020277
**Jensen R.H., et al. (2016)**	Denmark	Hospital 2013-2014	EUCAST EDef 7.2 /E-test	EUCAST	*C. tropicalis, C. krusei, C. parapsilosis*	FLU, AND	10.1128/AAC.01763-15
**Raja N.S. (2020)**	UK	Hospital 2006-2017	Germ tube/ Auxacolor 2 (Bio-Rad,, Marnes-la-Coquette, France)/API 20C Aux system /VITEK 2	Not Available	*C. albicans, C. glabrata, C. parapsilosis, C. tropicalis*	FLU,MIC,AMB	10.1111/ijcp.13655
**Rajendran R. et al.(2016)**	Switzerland	Hospital 2012-2013	broth microdiluition CLSI 2012	CLSI 2012	*C. albicans, C. glabrata, C. parapsilosis, C. tropicalis*	FLU, VOR, AND, AMB	10.3389/fmicb.2016.00915
**Risum M., et al.(2021)**	Switzerland	Hospital 2016-2018	EUCAST E.Def 7.3	EUCAST v. 10.0	*C. albicans, C. glabrata, C. parapsilosis, C. tropicalis, C. krusei*	FLU, AND, AMB	10.3390/jof7060491
**Spiers R., et al. (2018)**	United Kingdom	Laboratory 2007-2011	Vitek-2, API 32C, Sensititer Yeast One	CLSI (1997)M27A - M27S4.	*C. albicans, C. glabrata*	FLU, VOR, AMB, CAS	10.1093/mmy/myx165
**Vannini M., et al.(2022)**	France	Hospital 2014-2018	E-test (BioMerieux)	EUCAST /CLSI	*C. albicans, C. glabrata, C. parapsilosis* complex	FLU, AND, CAS, AMB	10.1016/j.mycmed.2021.101210
**Trouvé C., et al. (2017)**	Belgium	Hospital 2013-2014, 2003-2013	broth microdiluition EUCAST Edef 7.2	EUCAST	*C. albicans, C. glabrata, C. parapsilosis, C. tropicalis*	FLU, VOR, POS, AND, MIC, AMB	10.1007/s10096-016-2841-3
