# Supplementary material for: Tracking Candidemia Trends and Antifungal Resistance Patterns across Europe: An In-Depth Analysis of Surveillance Systems and Surveillance Studies
Source: J Fungi (Basel). 2024 Sep 29;10(10):685. doi: 10.3390/jof10100685 (PMC11514733; doi:10.3390/jof10100685)
Supplement: Supplementary file 1 [file jof-10-00685-s001.zip › Supplementary Table S4.pdf]

Supplementary Table S4. Number of isolates and resistance rates from different *Candida* species from all years of available national surveillance data in the respective countries for **a)** Azole resistances; **b)** Echinocandin resistances **c)** Polyene resistances. Number of resistant isolates out of all isolates (/) and resistance rates (in parenthesis) and the trends of resistant rates with β and p-values are shown. Arrows indicate the overall direction of the trend. Trends were calculated considering all data from 2015 to 2022, if data for ≥3 years were available. *N.A. trend was not calculated due to high variance.

a)

Azole-resistant *C. albicans*

**Country**	**Setting**	**2015**	**2016**	**2017**	**2018**	**2019**	**2020**	**2021**	**2022**	**Trend**
**Austria**	Hospital-wide	1/125 (0.8)	0/143 (0.0)	0/156 (0.0)	0/143 (0.0)	1/140 (0.7)	0/158 (0.0)	1/193 (0.5)	0/191 (0.0)	↔ (ß=-0.085; p=0.728)
**Croatia**	Hospital-wide	-	-	-	-	1/59 (1.7)	1/50 (2.0)	0/72 (0.0)	2/67 (3.0)	↔ (ß=0.128; p=0.793)
**Italy**	ICU	2/19 (10.5)	1/21 (4.8)	1/9 (11.1)	3/16 (18.8)	2/17 (11.8)	-	-	-	↔ (ß=0.150; p=0.368)
**Norway**	Hospital-wide	0/139 (0.0)	0/137 (0.0)	0/127 (0.0)	0/117 (0.0)	0/116 (0.0)	0/129 (0.0)	1/128 (0.8)	0/139 (0.0)	↔ (ß=0.645; p=0.263)
**Spain**	ICU	0/10 (0.0)	1/12 (8.3)	0/11 (0.0)	0/10 (0.0)	0/13 (0.0)	1/28 (3.6)	0/22 (0.0)	0/17 (0.0)	↔ (ß=-0.219; p=0.418)
**United Kingdom**	Hospital-wide	16/584 (2.7)	17/553 (3.1)	2/109 (1.8)	5/630 (0.8)	9/623 (1.4)	7/727 (1.0)	7/850 (0.8)	10/796 (1.3)	↘ **(ß=-0.174; p=0.014)**

Azole-resistant *C. glabrata*

Country	Setting	2015	2016	2017	2018	2019	2020	2021	2022	Trend
**Austria**	Hospital-wide	4/40 (10.0)	1/31 (3.2)	2/56 (3.6)	7/55 (12.7)	4/63 (6.3)	8/62 (12.9)	9/89 (10.1)	12/87 (13.8)	↔ (ß=0.110; p=0.140)
**Croatia**	Hospital-wide	-	-	-	-	28/28 (100.0)	27/27 (100.0)	39/39 (100.0)	28/28 (100.0)	N.A.*
**Norway**	Hospital-wide	9/35 (25.7)	6/35 (17.1)	14/36 (38.9)	7/33 (21.2)	9/29 (31.0)	3/24 (12.5)	4/36 (11.1)	7/41 (17.1)	↔ (ß=-0.083; p=0.228)
**United Kingdom**	Hospital-wide	99/286 (34.6)	144/332 (43.4)	13/30 (43.3)	39/367 (10.6)	32/285 (11.2)	25/268 (9.3)	36/302 (11.9)	54/372 (14.5)	↘ **(ß=-0.215; p=0.017)**

Azole-resistant *C. parapsilosis*

Country	Setting	2015	2016	2017	2018	2019	2020	2021	2022	Trend
**Austria**	Hospital-wide	3/25 (12.0)	4/23 (17.4)	1/23 (4.3)	0/18 (0.0)	0/26 (0.0)	0/18 (0.0)	2/27 (7.4)	0/28 (0.0)	↔ (ß=-0.361; p=0.130)
**Croatia**	Hospital-wide	-	-	-	-	40/52 (76.9)	28/38 (73.7)	64/80 (80.0)	54/67 (80.6)	↔ (ß=0.022; p=0.250)
**Norway**	Hospital-wide	1/8 (12.5)	1/15 (6.7)	1/10 (10.0)	0/7 (0.0)	0/22 (0.0)	2/6 (33.3)	2/14 (14.3)	0/17 (0.0)	↔ (ß=-0.045; p=0.847)
**Spain**	ICU	-	-	-	-	1/8 (12.5)	-	6/19 (31.6)	10/23 (43.5)	↔ (ß=0.384; p=0.085)
**United Kingdom**	Hospital-wide	2/135 (1.5)	2/149 (1.3)	0/44 (0.0)	4/199 (2.0)	4/186 (2.2)	4/192 (2.1)	10/250 (4.0)	4/218 (1.8)	↔ (ß=0.114; p=0.139)

Azole-resistant *C. tropicalis*										
**Country**	**Setting**	**2015**	**2016**	**2017**	**2018**	**2019**	**2020**	**2021**	**2022**	**Trend**
**Austria**	Hospital-wide	1/13 (7.7)	0/14 (0.0)	0/10 (0.0)	0/16 (0.0)	1/15 (6.7)	1/12 (8.3)	0/13 (0.0)	1/17 (5.9)	↔ (ß=0.070; p=0.705)
**Croatia**	Hospital-wide	-	-	-	-	0/2 (0.0)	1/5 (20.0)	0/5 (0.0)	2/10 (20.0)	↔ (ß=0.315; p=0.618)
**Norway**	Hospital-wide	0/9 (0.0)	0/13 (0.0)	0/15 (0.0)	0/8 (0.0)	1/17 (5.6)	0/15 (0.0)	0/13 (0.0)	0/12 (0.0)	↔ (ß=0.073; p=0.867)

b)

Echinocandin-resistant *C. albicans*										
**Country**	**Setting**	**2015**	**2016**	**2017**	**2018**	**2019**	**2020**	**2021**	**2022**	**Trend**
**Austria**	Hospital-wide	0/104 (0.0)	1/130 (0.8)	2/54 (3.7)	5/140 (3.6)	9/140 (6.4)	15/158 (9.5)	14/193 (7.3)	11/191 (5.8)	↗ **(ß= 0.230; p= 0.047)**
**Croatia**	Hospital-wide	-	-	-	-	1/59 (1.7)	1/50 (2.0)	1/72 (1.4)	0/67 (0.0)	↔ (ß= -0.480; p= 0.298)
**Norway**	Hospital-wide	2/139 (1.4)	0/137 (0.0)	0/127 (0.0)	1/117 (0.9)	0/116 (0.0)	1/129 (0.8)	0/128 (0.0)	1/139 (0.7)	↔ (ß= -0.297; p= 0.264)
**Spain**	ICU	0/7 (0.0)	0/10 (0.0)	0/7 (0.0)	0/7 (0.0)	1/8 (12.5)	0/22 (0.0)	0/10 (0.0)	0/10 (0.0)	↔ (ß= 0.018; p= 0.976)
**United Kingdom**	Hospital-wide	0/445 (0.0)	13/414 (3.1)	0/102 (0.0)	8/489 (1.6)	4/401 (1.0)	7/494 (1.4)	8/566 (1.4)	0/518 (0.0)	↔ (ß= -0.078; p= 0.576)

Echinocandin-resistant *C. glabrata*										
**Country**	**Setting**	**2015**	**2016**	**2017**	**2018**	**2019**	**2020**	**2021**	**2022**	**Trend**
**Austria**	Hospital-wide	0/39 (0.0)	2/26 (7.7)	0/52 (0.0)	0/58 (0.0)	1/81 (1.2)	3/69 (4.3)	0/89 (0.0)	0/87 (0.0)	↔ (ß= -0.149; p= 0.639)
**Croatia**	Hospital-wide	-	-	-	-	0/27 (0.0)	0/28 (0.0)	1/39 (2.6)	0/28 (0.0)	↔ (ß= 0.408; p= 0.718)
**Norway**	Hospital-wide	1/35 (2.9)	0/35 (0.0)	1/36 (2.8)	0/33 (0.0)	0/29 (0.0)	0/24 (0.0)	0/36 (0.0)	2/41 (4.9)	↔ (ß= 0.087; p= 0.690)

**United Kingdom**	Hospital-wide	4/258 (1.6)	10/299 (3.3)	0/48 (0.0)	11/334 (3.3)	16/248 (6.5)	23/249 (9.2)	23/256 (9.0)	13/325 (4.0)	↔ (ß= 0.151; p= 0.105)

Echinocandin-resistant *C. parapsilosis*

**Country**	**Setting**	**2015**	**2016**	**2017**	**2018**	**2019**	**2020**	**2021**	**2022**	**Trend**
**Austria**	Hospital-wide	2/23 (8.7)	1/21 (4.8)	0/23 (0.0)	2/18 (11.1)	0/25 (0.0)	2/18 (11.1)	6/27 (22.2)	1/28 (3.7)	↔ (ß= 0.116; p= 0.484)
**Croatia**	Hospital-wide	-	-	-	-	4/52 (7.7)	6/38 (15.8)	16/80 (20.0)	1/67 (1.6)	↔ (ß= -0.125; p= 0.798)
**Norway**	Hospital-wide	2/8 (25.0)	0/15 (0.0)	0/10 (0.0)	0/7 (0.0)	0/22 (0.0)	0/6 (0.0)	0/14 (0.0)	0/17 (0.0)	-*
**Spain**	ICU	-	-	-	-	6/7 (85.7)	-	0/16 (0.0)	2/13 (15.4)	↔ (ß= -0969; p= 0.376)
**United Kingdom**	Hospital-wide	2/118 (1.7)	6/116 (5.2)	0/40 (0.0)	2/164 (1.2)	2/139 (1.4)	0/156 (0.0)	0/191 (0.0)	1/155 (0.6)	↔ (ß= -0.361; p= 0.058)

Echinocandin-resistant C. tropicalis

**Country**	**Setting**	**2015**	**2016**	**2017**	**2018**	**2019**	**2020**	**2021**	**2022**	**Trend**
**Austria**	Hospital-wide	1/13 (7.7)	0/14 (0.0)	0/8 (0.0)	0/15 (0.0)	2/15 (13.3)	1/12 (8.3)	0/13 (0.0)	1/17 (5.9)	↔ (ß= 0.062; p= 0.748)
**Croatia**	Hospital-wide	-	-	-	-	0/2 (0.0)	0/6 (0.0)	0/5 (0.0)	0/10 (0.0)	↔ (ß= -0.368; p= 0.634)

**Norway**	Hospital-wide	0/9 (0.0)	0/13 (0.0)	1/15 (6.7)	0/8 (0.0)	0/17 (0.0)	0/15 (0.0)	0/13 (0.0)	0/12 (0.0)	↔ (ß= -0.371; p= 0.430)

**Echinocandin-resistant *C. krusei***										
**Country**	**Setting**	**2015**	**2016**	**2017**	**2018**	**2019**	**2020**	**2021**	**2022**	**Trend**
**Croatia**	Hospital-wide	-	-	-	-	0/7 (0.0)	0/9 (0.0)	0/4 (0.0)	0/6 (0.0)	↔ (ß= 0.109; p= 0.879)

c)

Polyene-resistant *C. albicans*										
**Country**	**Setting**	**2015**	**2016**	**2017**	**2018**	**2019**	**2020**	**2021**	**2022**	**Trend**
**Austria**	Hospital-wide	0/105 (0.0)	0/143 (0.0)	0/156 (0.0)	0/143 (0.0)	0/133 (0.0)	0/116 (0.0)	0/193 (0.0)	0/191 (0.0)	↔ (ß= -0.056; p= 0.762)
**Croatia**	Hospital-wide	-	-	-	-	0/59 (0.0)	0/50 (0.0)	0/72 (0.0)	0/67 (0.0)	↔ (ß= -0.073; p= 0.918)
**Norway**	Hospital-wide	0/139 (0.0)	0/137 (0.0)	0/127 (0.0)	0/117 (0.0)	0/116 (0.0)	0/129 (0.0)	0/128 (0.0)	0/15 (0.0)	↔ (ß= 0.181; p= 0.403)
**Spain**	ICU	0/7 (0.0)	1/12 (8.3)	0/9 (0.0)	0/10 (0.0)	0/12 (0.0)	1/26 (3.8)	0/20 (0.0)	0/139 (0.0)	↔ (ß= -0.426; p= 0.167)
**United Kingdom**	Hospital-wide	7/483 (1.4)	4/488 (0.8)	0/96 (0.0)	3/563 (0.5)	6/520 (1.2)	9/604 (1.5)	6/726 (0.8)	5/697 (0.7)	↔ (ß= -0.031; p= 0.641)

Polyene-resistant *C. glabrata*										
**Country**	**Setting**	**2015**	**2016**	**2017**	**2018**	**2019**	**2020**	**2021**	**2022**	**Trend**
**Austria**	Hospital-wide	0/37 (0.0)	0/31 (0.0)	0/56 (0.0)	0/62 (0.0)	1/77 (1.3)	0/50 (0.0)	0/89 (0.0)	0/87 (0.0)	↔ (ß= -0.031; p= 0.944)
**Croatia**	Hospital-wide	-	-	-	-	0/28 (0.0)	0/27 (0.0)	0/39 (0.0)	0/28 (0.0)	↔ (ß= -0.036; p= 0.961)
**Norway**	Hospital-wide	0/35 (0.0)	0/35 (0.0)	0/36 (0.0)	0/33 (0.0)	0/29 (0.0)	0/24 (0.0)	0/36 (0.0)	0/41 (0.0)	↔ (ß= 0.001; p= 0.996)

**United Kingdom**	Hospital-wide	9/283 (3.2)	3/345 (0.9)	0/52 (0.0)	4/465 (0.9)	1/403 (0.2)	3/385 (0.8)	0/410 (0.0)	8/452 (1.8)	↔ (ß= -0.138; p= 0.395)

Polyene-resistant *C. parapsilosis*

**Country**	**Setting**	**2015**	**2016**	**2017**	**2018**	**2019**	**2020**	**2021**	**2022**	**Trend**
**Austria**	Hospital-wide	0/24 (0.0)	0/23 (0.0)	0/23 (0.0)	0/18 (0.0)	0/24 (0.0)	0/16 (0.0)	0/27 (0.0)	0/28 (0.0)	↔ (ß= -0.013; p= 0.943)
**Croatia**	Hospital-wide	-	-	-	-	0/52 (0.0)	0/38 (0.0)	0/80 (0.0)	1/67 (1.5)	-*
**Norway**	Hospital-wide	0/8 (0.0)	0/15 (0.0)	0/10 (0.0)	0/7 (0.0)	0/22 (0.0)	0/6 (0.0)	0/14 (0.0)	0/17 (0.0)	↔ (ß= -0.051; p= 0.784)
**Spain**	ICU	-	-	-	-	0/8 (0.0)	-	0/15 (0.0)	0/22 (0.0)	↔ (ß= -0.309; p= 0.766)
**United Kingdom**	Hospital-wide	1/128 (0.8)	3/140 (2.1)	2/38 (5.3)	0/164 (0.0)	0/169 (0.0)	1/169 (0.6)	0/215 (0.0)	1/205 (0.5)	↔ (ß= -0.306; p= 0.233)

Polyene-resistant *C. tropicalis*

**Country**	**Setting**	**2015**	**2016**	**2017**	**2018**	**2019**	**2020**	**2021**	**2022**	**Trend**
**Austria**	Hospital-wide	0/12 (0.0)	0/14 (0.0)	0/10 (0.0)	0/16 (0.0)	0/14 (0.0)	0/11 (0.0)	0/13 (0.0)	0/17 (0.0)	↔ (ß= -0.025; p= 0.893)
**Croatia**	Hospital-wide	-	-	-	-	0/2 (0.0)	0/6 (0.0)	0/5 (0.0)	2/10 (20.0)	-*

**Norway**	Hospital-wide	0/9 (0.0)	0/13 (0.0)	0/15 (0.0)	0/8 (0.0)	0/17 (0.0)	0/15 (0.0)	0/13 (0.0)	0/12 (0.0)	↔ (ß= -0.030; p= 0.878)

Polyene-resistant *C. krusei*

Country	Setting	2015	2016	2017	2018	2019	2020	2021	2022	Trend
**Croatia**	Hospital-wide	-	-	-	-	0/7 (0.0)	7/9 (77.8)	1/4 (25.0)	3/6 (50.0)	↔ (ß= 0.188; p= 0.713)
